# The effects of an Audio Visual Assisted Therapy Aid for Refractory auditory hallucinations (AVATAR therapy): study protocol for a randomised controlled trial

**DOI:** 10.1186/s13063-015-0888-6

**Published:** 2015-08-13

**Authors:** Tom K.J Craig, Mar Rus-Calafell, Thomas Ward, Miriam Fornells-Ambrojo, Paul McCrone, Richard Emsley, Philippa Garety

**Affiliations:** Department of Health Service and Population Research, Institute of Psychiatry, Psychology and Neuroscience, King’s College London, London, UK; Department of Psychology, Institute of Psychiatry, Psychology and Neuroscience, King’s College London, London, UK; Research Department of Clinical, Educational and Health Psychology University College London, London, UK; Centre for Biostatistics, Institute of Population Health, The University of Manchester, Manchester Academic Health Science Centre, London, UK

**Keywords:** Auditory hallucinations, Voices, Avatar, Psychosis, Therapy

## Abstract

**Background:**

Psychological interventions which adopt an explicitly interpersonal approach are a recent development in the treatment of distressing voices. AVATAR therapy is one such approach which creates a direct dialogue between a voice-hearer and a computerised representation of their persecutory voice (the avatar) through which the person may be supported to gain a sense of greater power and control. The main objective of the trial is to test the clinical efficacy of this therapy to reduce the frequency and severity of auditory verbal hallucinations (AVH). Secondary objectives of the study are to explore explanatory mechanisms of action and potential moderators, to carry out a qualitative evaluation of participants’ experience and to conduct an economic evaluation.

**Methods/Design:**

The AVATAR randomised clinical trial will independently randomise 142 participants to receive either 7 sessions of AVATAR therapy or supportive counselling (SC). The study population will be individuals with schizophrenia spectrum and other psychotic disorders who report hearing persistent distressing voices, for more than 12 months, which are unresponsive or only partially responsive to antipsychotic medication. The main hypotheses are that, compared to SC, AVATAR therapy will reduce the frequency and severity of AVH and will also reduce the reported omnipotence and malevolence of these voices. Assessments will occur at 0 weeks (baseline), 12 weeks (post-intervention) and 24 weeks (follow-up), and will be carried out by blinded assessors. Both interventions will be delivered in a community-based mental health centre. Therapy competence and adherence will be monitored in both groups. Statistical analysis will follow the intention-to-treat principle and data will be analysed using a mixed (random) effects model at each post treatment time point separately. A formal mediation and moderator analysis using contemporary causal inference methods will be conducted as a secondary analysis. The trial is funded by the Welcome Trust (WT).

**Discussion:**

AVATAR therapy showed promising effects in a pilot study, but the efficacy of the approach needs to be examined in a larger randomised clinical trial before wider dissemination and implementation in mental health services.

**Trial registration:**

Current Controlled Trials ISRCTN: 65314790, registration date: 27 March 2013.

## Background

Previous studies have shown that around 70 % of people with schizophrenia report auditory hallucinations [1]. Voice hearing or auditory verbal hallucination (AVH) is the most commonly reported form of auditory hallucinations [2, 3] and is typically defined as hearing a voice or other sound in the absence of an external stimulus [4]. These AVHs frequently provoke high levels of distress and interference in the daily lives of those who experience them and, therefore, have become a major target of psychological therapies for psychosis [5].

**Fig. 1:**
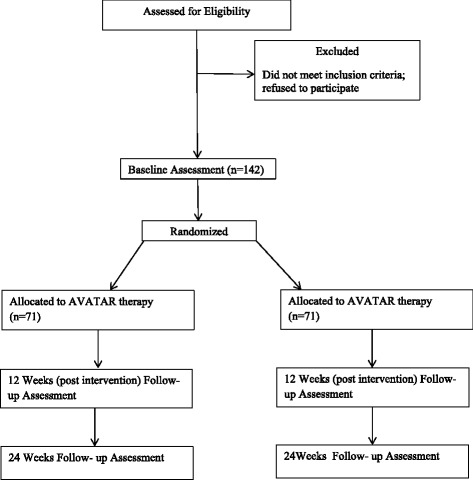
Study flowchart

**Fig. 2:**
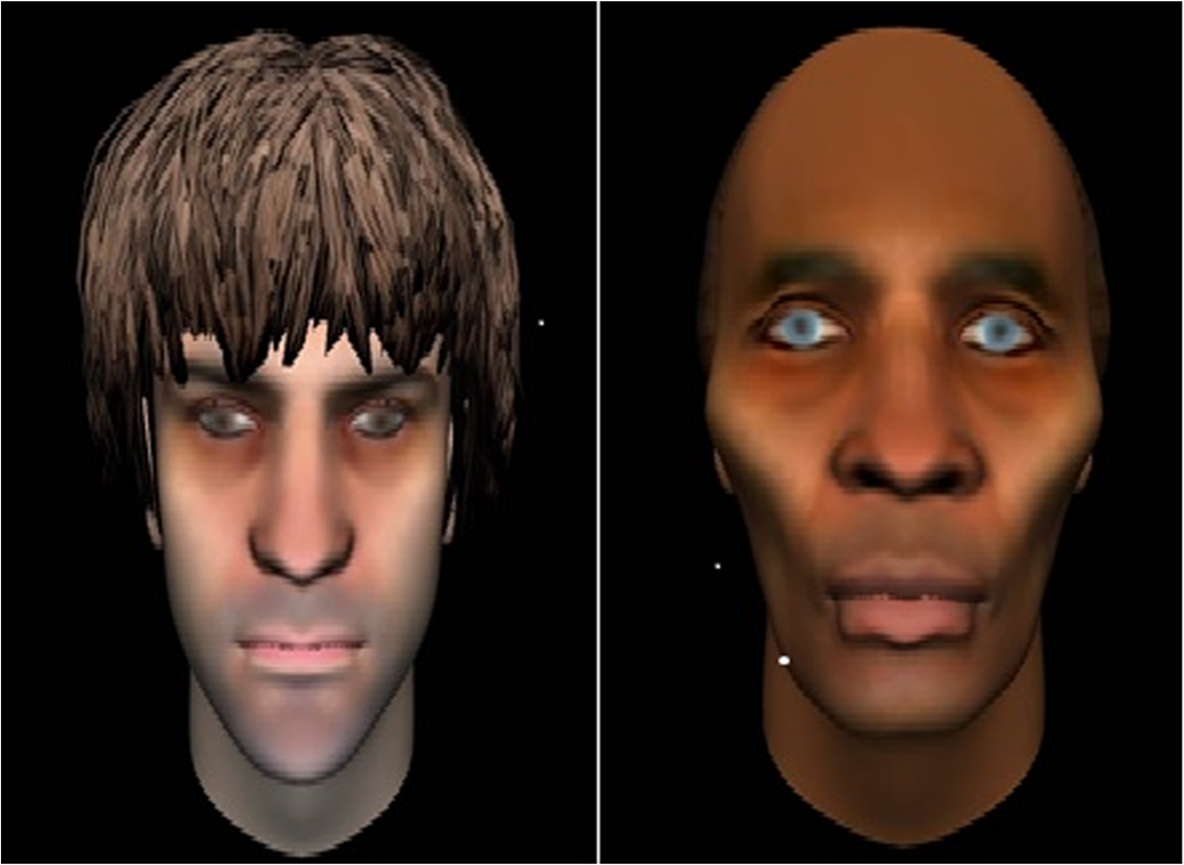
Examples of avatars

Drawing on seminal early work from Chadwick and Birchwood, cognitive models of voice- hearing propose that beliefs about voices (specifically regarding identity, power, intention and control) are key to understanding distress and maladaptive responding [6, 7]. In their model, Morrison and colleagues specifically propose that auditory hallucinations occur when a person misattributes an internal experience (e.g. intrusive thought) to an external source [8]. It is argued that subsequent maladaptive appraisal processes are maintained by safety behaviours (including selective attention), faulty self-knowledge (including metacognition), social knowledge, mood and physiology [9]. Birchwood and colleagues [10, 11] developed their model by applying social rank theory [12, 13] to voice-hearing and found that individuals who experienced powerlessness and inferiority in social relationships were more likely to report similar experiences during the voice interaction [10, 14]. It is argued that early powerlessness and perceived inferiority in social relationships establish social schemata that drive subsequent appraisals of voices leading, in turn, to significant levels of distress and depression [11]. Recent reviews have provided support for the proposal that social schema may mediate the appraisal-distress relationship with the implication that therapies could benefit from targeting social and interpersonal variables [2, 14].

These theoretical developments have informed a specific cognitive therapy for command hallucinations [15, 16]. A randomised controlled trial of this approach [15] has recently reported a reduction in the rate of compliance compared with the treatment as usual group (odds ratio 0·45) along with an associated reduction in the specific treatment target (the power difference between the perceived threat of the voice and the hearer’s ability to mitigate this threat). Other approaches have also adopted an explicitly relational approach, targeting key aspects of the voice relationship including appraisals of relative power and assertiveness [17, 18].

More recently, AVATAR therapy has been developed as a relational approach by Professor Julian Leff [19, 20]. This builds on the previous theoretical and clinical developments within the context of a novel therapeutic milieu. The main goal of this therapy is to facilitate a dialogue between the patient and a computerised representation of their persecutory voice in which the voice hearer is assisted to gain control over the distressing voice. The approach uses computer technology developed by the Speech, Hearing and Phonetics Department at University College London which enables each participant to create a visual representation of the entity (human or non-human) that they believe is talking to them. Additional software is used to transform the voice of the therapist to match closely the pitch and tone of the voice the patient reports hearing, the two processes finally being combined to produce a computer simulation (a virtual agent or ‘avatar’) through which the therapist can interact with the participant. The therapist promotes a dialogue between the participant and the avatar in which the avatar progressively comes under the participant’s control. The sessions are audio- recorded and provided to the participant on an MP3 player for continued use at home [19].

### Pilot study

In an initial pilot study, comparing the therapy with a waiting list control group, a maximum of 7 sessions lasting 30 minutes resulted in highly significant reductions in the participants’ hallucinations and associated distress, as well as a reduction in scores on the omnipotence and malevolence subscales in the Revised version of the Beliefs about Voices Questionnaire (BAVQ-R) [19].

Researchers in the pilot study asked the participants how close a match there was between the image on the monitor or the voice they had selected and what they believed their persecutor looked and sounded like. Ratings of the match were not reported or explored as possible treatment moderators but two participants said the avatar was not a good representation of their hallucination [20]. In terms of feasibility, of the twenty-seven people referred to the trial, one declined consent, four withdrew before therapy commenced and five did not complete the total course of the therapy.

In light of the encouraging results, the next step is to test the therapy’s efficacy in a larger methodologically rigorous clinical trial, in which a comparison is made between AVATAR therapy and a control condition, in order to take account of non-specific elements of therapy exposure, before wider dissemination of the therapy approach.

While the pilot did not include a formal mediation analysis, putative mechanisms emerge from the key treatment ‘phases’. Within the first phase the participants’ key task is to develop assertiveness with the avatar’s character gradually changing to become conciliatory or even helpful in line with the increasing strength and confidence of the person. Commonalities with Cognitive Therapy for Command Hallucinations [16, 15] and other relational approaches [17] along with the pilot findings, suggest that changes in beliefs about voices (specifically related to omnipotence and malevolence) and appraisal of the voice relationship (specifically relative power and assertiveness) are likely mechanisms of action. The second phase specifically targets improvements in self-concept and development of a more positive identity, work that is consistent with recent approaches emphasising the importance of self-esteem and self-compassion in working with distressing voices [21, 22]. Finally, given that anxiety processes are seen as central in the maintenance of distressing voices [9] and that AVATAR therapy involves an exposure to a distressing stimulus (voice content and image), reductions in anxiety may also be an important mechanism of action. With regards to potential moderators, engagement with, and therapeutic response to, cognitive behaviour therapy for psychosis have been predicted by baseline beliefs about illness [23] and social factors such as presence of a caregiver [24]. Given the novelty of the set-up AVATAR therapy may also have more specific treatment moderators. The task of creating a single avatar to represent the person’s voice experience, incorporating discernible verbatim content, suggests that the number of voices, type of content and ratings of the created voice/image may act as additional moderators.

### Research objectives and hypotheses

The trial has three main objectives:

1. To test the clinical efficacy of the therapy compared to supportive counselling (SC).
2. To determine preliminary estimates of cost-effectiveness of the AVATAR therapy.
3. To explore explanatory mechanisms of action as well as moderators for AVATAR therapy.

The trial hypotheses are:

- AVATAR therapy will be more effective in reducing the frequency and severity of auditory hallucinations, in comparison to SC.
- AVATAR therapy will be more effective in reducing the reported omnipotence and malevolence of auditory hallucinations, in comparison to SC.
- The improvements attributable to AVATAR therapy will be maintained at 24 weeks follow-up.
- AVATAR therapy will be more cost-effective than SC.
- The mediators of treatment effects for AVATAR therapy on changes in auditory hallucinations will be beliefs about voices (specifically omnipotence and malevolence), beliefs about the self (improved self-esteem), appraisal of voice relationship (specifically relative power and assertiveness), and reduction in anxiety.

The moderators of the treatment effects for AVATAR therapy will be number of voices, type of content (derogatory versus non-derogatory), ratings of the created voice/image, beliefs about problems and social support.

## Methods/Design

The study design is a single blind randomised controlled trial. Patients who meet inclusion criteria (see below) will be independently randomised to receive either 7 sessions of AVATAR therapy or SC (see Fig. 1). Both groups will continue to receive standard psychiatric care. Written consent will be obtained from each eligible participant prior to assessment and randomisation. Participants will be allocated to conditions using randomised permuted blocks (with a block size randomly varying between 2 and 6). The block sizes will not be disclosed to ensure allocation concealment. Randomisation will be carried out at the point of consent through an independent web-based service provided by the UKCRC Registered Clinical Trials Unit (CTU) at King’s College London (Registration Number 053). The randomisation will be conducted by the CTU in order to keep the data management and the statistician blind to the study condition. Assessments will be conducted by research assessors blind to therapy allocation. In case of unblinding, the trial coordinator will switch assessors for follow-up assessments. The reliability of the raters on the assessment battery will be formally assessed. For reporting the trial, the Consolidated Standards of Reporting Trials (CONSORT) with the extension for non-pharmacologic treatment and the Recommendations for Interventional Trials (SPIRIT) guidelines will be followed [25]. All data collected and study-related information will be stored securely at the Institute of Psychiatry, Psychology and Neuroscience, King’s College, London.

### Participants

The inclusion criteria are as follows: 1) aged over 18 years; 2) have experienced troubling auditory hallucinations for at least 12 months; 3) primary diagnosis of non-organic psychosis (including *International Classification of Diseases* (ICD)-10 categories F20-29 and F30-39, subcategories with psychotic symptoms). Criteria for exclusion are as follows: 1) unable to give informed consent; 2) currently in receipt of cognitive behaviour therapy for psychosis or attending a group specific to hearing voices; 3) unable to identify a single dominant voice to work on; 4) refusing all medication; 5) a diagnosis of organic brain disease; 6) a primary substance dependency; 7) auditory hallucinations in a language not spoken by the therapists; 8) a command of spoken English inadequate for engaging in therapy; 9) inability to tolerate the assessment process.

Clinical staff will be informed of the study and basic criteria for inclusion and asked to refer patients who express an interest in taking part. Patients are also able to self-refer in response to information given to primary care centres and in response to posters in the relevant clinical areas of the Trust. The majority of the referrals will be received from the South London and Maudsley National Health Service (NHS) Foundation Trust. However, for the patients who are not currently within South London and Maudsley (SLaM) services, we will follow Patient Identification Centre (PICs: Health Boards or Trusts, which can identify possible participants for the study) activity procedure. Finally, other clinicians working in Mental Health Services in the UK who are aware of the study via clinical contacts or conferences conducted by the research team can also refer participants. All referrals will be screened for eligibility. Participants’ diagnosis will be confirmed by an independent experienced and trained psychiatrist examining medical case notes and using the OPCRIT system [26].

### Planned interventions

Eligible participants will be randomised to either the AVATAR therapy or SC arm of the trial. AVATAR therapy and SC will both be delivered over 7 (1 introductory session plus 6 therapy) sessions lasting approximately 45 minutes. Both therapies will be manualised and trial therapists provided with training and on-going supervision. Therapy sessions will be audio-recorded and assessed for therapy competence and adherence to the treatment model. Participants allocated to SC will have the opportunity to receive AVATAR therapy at the end of the trial.

### AVATAR therapy

Participants first create a computerised representation of the person or entity that they feel represents the source of their voices (see Fig. 2). Over the following sessions the therapist facilitates a direct dialogue between the person and the avatar they have created, in which the therapist voices the avatar’s speech. The system is set up in two rooms in the same building with two linked computers. Participants sit in one room facing the monitor on which the avatar appears. The therapist sits in a second room facing the monitor with a control panel that allows them to talk to the participant in their own voice or in the morphed avatar voice. The therapist can see and hear everything that is appearing on the participant’s monitor as well as the participant’s responses, adjusting therapeutic interventions and response to the avatar according to the unfolding dialogue. Should the participant be distressed at any point they can press a button on the desk that will terminate the session, replacing the on-screen avatar with a pleasant scene. The therapist also uses his/her judgement to anticipate distress and modify the avatar interaction accordingly. For more details on the software used in AVATAR therapy please see Leff et al. [19].

After completing the creation of the avatar (introductory session), the therapy is delivered in 6 weekly 45-minute sessions of which up to 20 minutes involves face-to-face work with the avatar and the remaining time is spent with the therapist and participant in direct contact both assessing current voices and planning the AVATAR session and subsequently debriefing on it. The number and progress of sessions is determined by a discussion with the participant at each session concerning any change in severity, content, malevolence or frequency of the hallucinations. Therapy will be terminated before six sessions if the participant has reported complete absence of any voices for at least three consecutive sessions. The total number of sessions can be extended by up to three further sessions where there is a clear rationale for the likely benefit of additional sessions, such as evidence of delayed and ongoing improvements during later sessions, on self-reported severity, content, malevolence or frequency of the voice (any additional sessions are agreed by consensus within the therapy team). Evidence of any adverse reactions will result in completion of an adverse events form (see safety assessment section below). Evidence of any adverse reactions to therapy will be closely monitored. Therapy will be terminated in the event of any significant deterioration in mental state which renders the continuation of therapy inadvisable (for example where the therapy sessions are associated with significant increased distress and/or risk of harm to self/others); and any such termination would be decided through agreement between therapist (in consultation with the AVATAR therapy team), the participant and the relevant clinical team. All therapists delivering AVATAR therapy have at least 5 years of clinical experience in psychosis and have been trained by Professor Julian Leff on delivery of the therapy.

### Supportive counselling

The control condition comprises a manual-based, face-to-face, SC approach based on the manual used in the SOCRATES clinical trial [27] to control for non-specific elements of therapy exposure. It will be delivered over the same number and duration of sessions (including application of the same rules for early termination and extension as above), with the aim of matching the duration of total therapist contact time to that in the AVATAR arm.

### Audio-recordings

All therapy sessions are audio-recorded, with participants’ consent. The participant is provided with an audio-recording of the session on an MP3 player with instructions to listen at times of their choosing between sessions (for those in the AVATAR arm the recording is the dialogue with the avatar, for those in the SC arm the recording is a summary of the key points from the session).

### Assessments and follow-up

There are 3 assessment points: at baseline before randomisation, at 12 and 24 weeks. All measures apart from weekly in-session measures will be carried out by trained research staff. Participants will complete a number of self-completed and interview-based measures to assess the impact of interventions on outcomes and to explore potential mediators and moderators of therapy. All the assessment sessions will be audio-recorded to ensure the accuracy of data collection. The participants will be reimbursed £20 plus any travel expenses for each assessment session in recognition of their time and other out-of-pocket expenses.

### Measures

The primary outcome measure of the study is the total score on the auditory hallucinations subscale of the Psychotic Symptoms Rating Scale (PSYRATS-AH [28]) at the 12-week follow-up. It is an interviewer-assessed measure of the frequency and duration of auditory hallucinations over an average week. The PSYRATS is specifically designed for use with people suffering from psychosis, with inter-rater reliability ranging from 0.79 to 1.00 and high validity when compared with similar psychiatric scales [28]. It has been extensively used in research with these populations and has been shown to be acceptable to service users.

Secondary outcome measures will include the Revised version of the Beliefs about Voices Questionnaire (BAVQ-R, [29]: a self-report measure which focuses on the patient’s beliefs about the voices, and indexes how likely the voices are to affect behaviour. In addition to the total score, changes in two subscales will be analysed: omnipotence and malevolence.

Other standardised measures will be used to assess changes in voice experience and appraisals: the Voice Acceptance and Action Scale: VAAS [30] and Voice Power Differential Scale: VPDS [10]. We will assess changes in delusions using the Psychotic Symptoms Rating Scale: PSYRATS-D [28] and other psychotic symptoms using the Scale for Assessment of Positive and Negative Symptoms: SAPS and SANS [31]. Other standardised questionnaires will assess anxiety and depression (Depression Anxiety and Stress Scale: DASS-21 [32]; (Calgary Depression Scale [33]); self-esteem [34]; quality of life (Short Assessment of Quality of Life, MANSA [35]); and illicit drug use [36].

In order to assess progress during the therapy, the PSYRATS-AH [28] and the VPDS [10] will be completed by the therapist and the participant at each therapy session. Additionally, an adapted version of the Sense of Presence Questionnaire [37], the State Social Paranoia Scale [38] in relation to the avatar along with analogical scales will be used to assess the participant’s interaction with the avatar and the match obtained during the creation of the image/voice. Burns Empathy Scale [39] will be completed in sessions 2, 4 and 6. All the questionnaires in the study are being delivered in paper-and-pencil format.

### Assessment of safety

Serious adverse events will be monitored and recorded throughout the study period, as well as complaints about therapy. The following are considered as adverse events: 1) hospital admissions; 2) home treatment team involvement; 3) suicide attempts; 4) any violent incident necessitating police involvement (whether victim or accused); 5) self-harming behaviour; 6) all deaths. Furthermore, the trial coordinator will review all participant clinical notes and contact clinicians for any important additional information. These events are reported to the Data Monitoring and Ethics Committee (DMEC) of the trial. Participants have the right to withdraw from the study at any time.

### Sample size

We will recruit 142 participants to the trial, approximately 71 in each arm. The sample size calculation is based on the primary outcome measure of a reduction in the severity of auditory hallucinations as measured by the total score of the Auditory Hallucinations component of the Psychotic Symptoms Rating Scale (PSYRATS). Compared with treatment as usual, a clinically meaningful change in the total PSYRATS score is 5 units, which corresponds to an effect size of approximately *d* = 0.8, whereas supportive therapy typically achieves a modest effect of *d* = 0.2. Our sample size of 71 in each group will have 90 % power to detect an effect size of 0.6 using a two-group *t* test with a 0.05 two-sided significance level while also allowing for a 20 % loss to follow-up. In practice, the power will be increased by using a mixed (random) effects model allowing for baseline covariates (rather than a simple *t* test) to gain precision in the effect estimates.

### Analysis

#### Statistical analysis

Analysis will be conducted in Stata version 13 (StataCorp, College Station, TX, USA) [40]. Descriptive statistics within each randomised group will be presented for baseline values. These will include counts and percentages for binary and categorical variables and means and standard deviations, or medians with lower and upper quartiles, for continuous variables, along with minimum and maximum values and counts of missing values. There will be no tests of statistical significance or confidence intervals for differences between randomised groups on any baseline variable.

The primary hypothesis for change in the severity of auditory hallucinations as measured by the total PSYRATS-AH score will be analysed using a mixed (random) effects model allowing for the baseline measurement of PSYRATS-AH and treatment assignment as fixed effects, at 12 weeks. This takes account of missing outcomes assuming a missing at random mechanism which allows missingness to depend on baseline severity of AH and treatment assignment. Therapist effects will be modelled by including a random effect for each therapist in the two therapy conditions. The use of a mixed (random) effect models will allow for estimation of the intra-cluster correlation coefficient, a measure of the proportion of variance in outcome because of therapist effects, which can be used in future applications; no estimate of this is currently available. Secondary outcome measures will be analysed using the same modelling approach. This includes analysis of the primary outcome and secondary outcomes at 24 weeks.

A secondary mediation analysis will investigate putative mediational factors (including beliefs about voices, self-esteem, anxiety/levels of distress) using modern causal inference methods. This involves using parametric regression models to test for mediation of the AVATAR therapy on AH through the putative mediators. Analyses will adjust for baseline measures of the mediator, outcomes, and possible measured confounders. We will include repeated measurement of mediators and outcomes to account for classical measurement error and baseline confounding, and instrumental variable methods (baseline covariate by randomisation interactions as potential instruments) to investigate the sensitivity of the estimates to these problems and that of unmeasured confounding [41].

Moderators will be assessed separately by repeating the primary analysis models and including interaction terms between the randomised intervention and each moderator. The coefficient of the interaction term is a measure of whether the treatment effect differs between levels of the moderator.

### Qualitative evaluation

A qualitative evaluation of participants’ experience of therapy will be carried out to explore the processes of implementation of the AVATAR therapy, and participants’ perception of the intervention as appropriate to addressing their auditory hallucinations and to explore barriers and facilitators of therapy. It will be based on an individual semi-structured interview using a topic guide. Questions will focus on the participants’ experience of using the computer software, their views on how ‘realistic’ they found the avatar to be and their opinion as to how important this is, the environment in which the intervention is offered and duration of sessions. Other external aspects related to the intervention, such as use of the MP3 players between sessions and facilitators to the delivery of the intervention, will be also explored. Participants will also be asked how their experience of therapy has affected their views about their voices, and their views about how well they will manage their voices in the future.

Independent qualitative interviews will be also conducted with the trial therapists in order to gather their impressions of conducting the sessions, the challenge of enacting the avatar, and their experience of group supervision. This will help refine therapies and for implementation purposes.

### Health economic analysis

Service use will be measured for a retrospective 3-month period at baseline and 12-week and 24-week follow-up using the Client Service Receipt Inventory [42]. Service use will be measured comprehensively and include services provided by the NHS, other health and social care agencies, the criminal justice system and informal carers. In-patient admissions and length of stay will be recorded for the entire study period. Appropriate unit costs will be attached to the utilisation data in order to generate service costs for each participant. Costs will be compared across the trial conditions and linked to the primary outcome measure. If costs are lower and outcomes better, then the intervention will be considered ‘dominant’. If costs are higher and outcomes better then incremental cost-effectiveness ratios will be reported and these will indicate the extra cost incurred to achieve an extra unit of outcome. Uncertainty around cost-effectiveness estimates will be explored using cost-effectiveness planes and cost-effectiveness acceptability curves. Both of these will be generated using 1000 bootstrapped re-samples from the data. To make sure all key elements of the economic evaluation are properly reported, the CHEERS checklist will be completed [43].

### Research ethics approval and governance

King’s College London is the research sponsor. The trial will be conducted in accordance with the principles of the Declaration of Helsinki (current 2013 version). The study has been reviewed and approved by the London-Hampstead Research Ethics Committee (+: 13/Lo/0482). Modifications to the protocol require a formal amendment to the protocol. This will be completed by the Primary Investigator and Trial Coordinator and approved by the London-Hampstead Research Ethics Committee. All changes and amendments to the protocol are supervised and approved by the sponsor. Medical Research Council Guidelines for Good Practice in Clinical Trials will be followed to ensure the trial integrity and participants’ safety and wellbeing [44]. The Trial Steering Committee (TSC) comprises one clinician independent chair, one senior clinician and a service user. A DMEC includes a clinician as independent chair, a senior clinician and statistician.

## Discussion

Around a quarter of people suffering from psychotic conditions continue to experience auditory hallucinations despite adequate drug treatment [45, 46]. Treatment of this problem is a public health priority because people suffering from distressing voices often have a low quality of life [47]. Recent developments in treatment for distressing voices, which focus on the interpersonal relationship between voice-hearer and their voice, include a specific cognitive therapy for command hallucinations [15], as well as other explicitly relational [48] and dialogic [49] approaches. AVATAR therapy offers a unique opportunity to work relationally, through real-time dialogue with an avatar, created by the hearer as a representation of their voice. Encouraging pilot data suggest that AVATAR therapy may represent an important and powerful new tool in the treatment of distressing voices [19].

The brevity of the therapy and its success in decreasing the frequency of the voices, their volume, and their negative impact on individuals’ lives requires both replication in a methodologically rigorous randomised clinical trial and an exploration of the possible mechanisms for these effects on experiences which have failed to respond adequately to antipsychotic medication [20]. Refinements to the software as well as the impact of the quality of the match between the creation (including voice and image) of the avatar are also underway. The trial is funded for 36 months. Final outcome assessment will be completed by mid-2016 and outcome results will become available by the end of the same year.

## Trial status

The trial started recruitment of participants in November 2013 and it will be open until early 2016.

## Abbreviations

AVH: auditory verbal hallucinations
AVATAR: Audio Visual Assisted Therapy Aid for Refractory auditory hallucinations
BAVQ-R: Beliefs about Voices Questionnaire Revised version
CONSORT: Consolidated Standards of Reporting Trials
CTU: Clinical Trials Unit
DASS-21: Depression Anxiety and Stress Scale
DMEC: Data Monitoring and Ethics Committee
NHS: National Health Service
ICD: *International Classification of Diseases*
PIC: Patient Identification Centre
PSYRATS: Psychotic Symptoms Rating Scale
SAPS/SANS: Scale for Assessment of Positive and Negative Symptoms
SC: supportive counselling
TSC: Trial Steering Committee
VAAS: Voice Acceptance and Action Scale
VPDS: Voice Power Differential Scale
WT: Welcome Trust
